# stPipe: a flexible and streamlined R/Bioconductor pipeline for preprocessing sequencing-based spatial transcriptomics data

**DOI:** 10.1093/nargab/lqaf167

**Published:** 2025-11-22

**Authors:** Yang Xu, Callum J Sargeant, Yue You, Yupei You, Shian Su, Changqing Wang, Luyi Tian, Yunshun Chen, Matthew E Ritchie

**Affiliations:** The Walter and Eliza Hall Institute of Medical Research, Parkville, VIC 3052, Australia; Department of Medical Biology, The University of Melbourne, Parkville, VIC 3010, Australia; The Walter and Eliza Hall Institute of Medical Research, Parkville, VIC 3052, Australia; Guangzhou National Laboratory, Guangzhou, 510005, China; GMU-GIBH Joint School of Life Sciences, Guangzhou Medical University, Guangzhou, 510182, China; The Walter and Eliza Hall Institute of Medical Research, Parkville, VIC 3052, Australia; Department of Medical Biology, The University of Melbourne, Parkville, VIC 3010, Australia; The Walter and Eliza Hall Institute of Medical Research, Parkville, VIC 3052, Australia; Department of Medical Biology, The University of Melbourne, Parkville, VIC 3010, Australia; The Walter and Eliza Hall Institute of Medical Research, Parkville, VIC 3052, Australia; Department of Medical Biology, The University of Melbourne, Parkville, VIC 3010, Australia; Guangzhou National Laboratory, Guangzhou, 510005, China; GMU-GIBH Joint School of Life Sciences, Guangzhou Medical University, Guangzhou, 510182, China; The Walter and Eliza Hall Institute of Medical Research, Parkville, VIC 3052, Australia; Department of Medical Biology, The University of Melbourne, Parkville, VIC 3010, Australia; The Walter and Eliza Hall Institute of Medical Research, Parkville, VIC 3052, Australia; Department of Medical Biology, The University of Melbourne, Parkville, VIC 3010, Australia

## Abstract

Spatial transcriptomics technology has developed rapidly in recent years, with various sequencing-based platforms such as 10× Visium, Slide-seq, and Stereo-seq becoming widely used by researchers. Each platform brings its own set of protocols and customized data analysis pipelines, which presents challenges when the goal is to obtain uniformly preprocessed data that is conveniently formatted for downstream analysis. To address the need for simpler, open-source solutions that deal with sequencing-based spatial transcriptomics (sST) data from different platforms, we present stPipe, a comprehensive and modular preprocessing pipeline for all current mainstream sST platforms. stPipe is implemented as an R/Bioconductor package that handles various analysis steps, including (i) data processing from raw FASTQ files to create a spatially resolved gene count matrix; (ii) the collation of relevant quality control metrics to ensure unwanted artifacts can be filtered; and (iii) the adoption of standardized data storage containers to allow results to be easily passed on to a wide range of downstream analysis packages. A key use case for stPipe is in methods benchmarking, and we demonstrate how the uniform processing of sST data collected on reference tissue samples from the *cadasSTre* and *SpatialBenchVisium* projects is made easier, allowing comparisons between different technology platforms and downstream analysis tools.

## Introduction

The rapid development of spatially resolved transcriptomics technology over the past 10 years has enabled a more comprehensive understanding of the patterns of gene expression and cellular communication across tissues [1]. Spatial transcriptomics (ST) has seen broad application in investigating tissue development, spatially resolved biomarker identification, cell state and fate determination across tissues in a wide range of research fields, including immunology, developmental biology, oncology, and neuroscience [2]. Various ST platforms that employ distinct methodologies have been developed. These can be broadly classified into two main categories: imaging-based spatial transcriptomics (iST) and sequencing-based spatial transcriptomics (sST), which differ fundamentally in their strategies for determining spatial localization and quantifying messenger RNA (mRNA) abundance [3]. Commercially available iST technologies such as Xenium (10× Genomics) and MERSCOPE (Vizgen) leverage fluorescent signatures and their intensity to measure target genes and their abundance via highly multiplexed single molecule fluorescence *in situ* hybridization. While imaging-based methods excel in spatial resolution, they are typically limited by the number of transcripts that can be simultaneously profiled [4]. In contrast, commercial sST platforms like Visium (10× Genomics) and Stereo-seq (BGI) depend on spatial barcodes arranged either deterministically or randomly on an array to reconstruct the spatial position of the target gene within the tissue. These sST methods then apply next-generation sequencing to the spatial libraries constructed to ascertain their expression level. Sequencing-based methods tend to sacrifice spatial resolution but allow genome-wide gene quantification.

10× Genomics Visium is one of the most popular and widely used ST platforms over the past 5 years [3]. 10× Genomics provides two Visium capture chemistries, which enable either fresh frozen (FF) tissue samples to be profiled using polyA-based capture of RNA or the analysis of formalin-fixed paraffin-embedded (FFPE) samples based on RNA templated ligation of pairs of gene target probes. Both of these approaches use slides covered with 55 μm-diameter spots with a center-to-center distance of 100 μm between horizontal spots [5]. For FFPE samples, pre-defined human (or mouse) transcriptome-centric probe-sets and spatial barcode allow lists enable Visium to capture both gene expression and spatial localization information simultaneously. For polyA-based FF samples, splicing-aware alignment of transcripts is performed for reads to map them to the reference genome. To accurately align ST sequencing data with the tissue image and perform pixel computation for each spot, fiducial alignment and tissue detection are required. To simplify the original Visium workflow, 10× Genomics developed the Visium CytAssist (CA) instrument to allow transfer of the tissue section from a standard glass slide onto the Visium slides, allowing the use of either pre-sectioned tissue or FFPE blocks [2]. The recently released Visium HD platform enables single cell-scale resolution via the tiled arrangement of millions of 2 × 2 μm barcoded squares that are typically binned (into 8 × 8 μm or 16 × 16 μm regions) for further analysis. This represents a greater than four-fold increase in spatial resolution over regular Visium technology. A practical example of where this enhanced resolution improves the biological insights gained was recently demonstrated in the study of normal colon mucosa, with Visium HD able to capture the characteristic hole pattern via the binning of barcoded squares, while the lower resolution Visium data could not recover this expected detail [6].

BGI’s DNA Nanoball (DNB) technology is used by Stereo-seq (SpaTial Enhanced REsolution Omics-sequencing) to recover data on the STOmics-GeneExpression Chip at up to nanometer level resolution [7]. DNB is generated by rolling circle amplification of circularized DNA oligonucleotides, forming spherical structures. Each DNB carries a 25-nt coordinate identity used to identify its spatial position during RNA capture and sequencing. The spatial location of each DNB is recorded with a diameter of 0.2 μm and a center-to-center distance of 0.5 μm to generate sub-cellular single-cell resolution data. Compared to 10× Visium technology, Stereo-seq can be analysed with less reliance on scRNA-seq data to perform deconvolution of the cell type mixtures that are present in data that is captured with courser resolution, such as Visium.

Slide-seq is another popular sST technology which uses Drop-seq like beads for oligonucleotide synthesis as spots with diameters of 10 μm that are randomly arranged on the array surface [8]. A recent updated version of this protocol (Slide-seq V2) modifies the library generation, bead synthesis, and array indexing steps to improve the mRNA capture sensitivity by ~10-fold over the original Slide-seq method [9]. Curio-seeker is the commercially available version of Slide-seq V2.

These three technologies differ significantly in their approaches to spatial barcode synthesis, which directly impacts data quality and the preprocessing strategies. 10× Visium uses chemical or optical methods to directly synthesize and attach barcodes to predefined grid points on the glass slide. Each grid point has a pre-designed unique barcode, which is consistent across different slides, facilitating standardized cross-experiment analysis. BGI Stereo-seq employs rolling circle amplification technology to amplify pre-designed barcodes into DNA nanoballs, which are densely packed on a solid surface through chemical bonding or electrostatic interactions. Each nanoball carries a unique barcode, distributed with ultra-high density and randomness. This approach supports higher spatial resolution but requires high-precision imaging techniques to decode barcode positions and obtain spatial information. Slide-seq uses chemical coupling reactions to attach pre-designed barcodes onto the surfaces of microbeads, ensuring that each microbead carries a unique barcode. These microbeads are randomly distributed on a substrate, such as a polyacrylamide gel or a glass surface. The spatial positions of the microbeads are resolved through high-resolution imaging techniques, such as fluorescence microscopy, and correlated with their respective barcode sequences. Barcode distribution is thus entirely random.

The systematic differences between sST technologies has led to the development of platform-specific data preprocessing tools, which handle the raw sequencing reads and summarize these into a per sample spatial gene count matrix. For instance, Space Ranger, developed by 10× Genomics, processes raw 10× Genomics Visium data alongside bright-field or fluorescence microscopy images [10]. The Stereo-seq Analysis Workflow, SAW, designed by BGI, facilitates the preprocessing of Stereo-seq data to generate spatial gene expression matrices [11]. The Curio Seeker bioinformatics pipeline developed by Curio Bioscience is written in Nextflow to support the preprocessing of sST data from the Curio Seeker spatial mapping kit [12]. Notably, this pipeline is compatible with both Singularity (Apptainer) and Docker environments, enabling flexible execution. In terms of open-source analysis solutions for sST data preprocessing, few solutions exist. Spacemake is one Python-based tool previously developed for Visium and Slide-seq data, but it lacks support for nanoball-based ST methods such as BGI Stereo-seq and cannot be seamlessly integrated with other R-based software packages [13].

To fill this gap, we developed the stPipe R package, which builds upon the foundations laid by our earlier scPipe package [14]. stPipe is the first fully integrated R package that can process paired-end (PE) sequencing reads from multiple sST platforms to create a spatial count matrix in preparation for downstream analysis. The output of stPipe is fully compatible with current popular mainstream tools for ST analysis available from various open-source projects, which facilitates a comprehensive beginning-to-end analysis of sST data. In this article, we introduce the main features and implementation details of stPipe and demonstrate its use in quality control (QC) and downstream analysis using various benchmarking datasets to compare different technologies.

## Materials and methods

### Spatial transcriptomics datasets analysed

#### 10× Visium mouse spleen dataset

The *SpatialBenchVisium* dataset [15] provides spatially resolved transcriptomic profiles of mouse spleens from 8-week-old mice post recovery from malaria infection. Samples are available across different combinations of 10× Visium sample handling and profiling protocols, including sample preparation protocols for FF at optimal cutting temperature or FFPE; tissue placement as direct tissue placement on the slide with the use of CA or manual placement. Samples can be grouped by sex (male or female) or genotype (knock-out, control, or wild type). The reads were sequenced by an Illumina NextSeq 2000 according to 10× guidelines and processed by stPipe. This dataset is available under GEO accession number GSE254652.

#### 10× Visium mouse brain dataset

This dataset originates from the publicly available 10× Genomics Visium platform and represents a coronal section of FFPE mouse brain tissue. Specifically, the sample comprises brain tissue obtained from 8-week-old male mice, oriented in the coronal plane, sectioned to a thickness of 5 μm, and mounted onto Visium Gene Expression slides in accordance with the standardized 10× Genomics Visium protocol. H&E imaging was performed using the Metafer Slide Scanning Microscope (MetaSystems), providing detailed histological context for subsequent spatial gene expression profiling.

#### Slide-seq V2 mouse brain dataset

Mouse hippocampus samples (Puck_191204_01) were analysed using stPipe as one of the use cases. A spline was fitted along the CA1 pyramidal cell layer, and beads were averaged to generate a gene expression profile extending ~100 μm into the basal neuropil and ~400 μm into the proximal neuropil [9]. Samples were then sequenced based on the Illumina NovaSeq platform. This dataset is available from the Broad Institute’s online single cell portal.

#### Stereo-seq mouse brain dataset

This Stereo-seq mouse brain dataset (sample id: SS200000135TL_D1) is publicly available in SRA via BioProject PRJNA1036005 and is used as a demo dataset in the SAW pipeline [11]. This sample contains over one billion reads from a chip size of 1 × 1 cm.

### Downstream analysis

#### Deconvolution and cell type annotation for mouse brain datasets

Deconvolution and cell type annotation were performed on three mouse brain datasets using integrated workflow of Seurat version 5.2 and spacer version 2.2.1 [16]. An existing and well-annotated mouse brain scRNA-seq dataset was used [17]. The deconvolution mode was set as doublet for Slide-seq and multi for Visium and Stereo-seq data as recommended in the spacer documentation.

#### Marker gene selection and cell type annotation for mouse spleen datasets

Marker genes for all cell types in the mouse spleen datasets (Supplementary Table S1) were collected in accordance to the previous literature [15]. The per-sample cluster scoring method was used carefully in line with the previous study. Specifically, the expression levels of predefined cell type marker gene sets were first summed across all spatial spots within each cluster. These aggregated values were then normalized by the number of spots in the corresponding cluster to account for differences in cluster size. This procedure was applied across all clusters and samples. Subsequently, a cluster score was computed as the log-fold change in marker gene expression between each cluster of interest and the combined set of all other clusters. Heatmap of cluster scores was created using pheatmap version 1.0.12.

#### Pseudo-bulk differential expression analysis of mouse spleen data

Statistical testing to identify differentially expressed genes was conducted using limma version 3.58.1 [18] and edgeR version 4.0.16 [19]. B cell clusters were subset from each mouse spleen dataset between male and female groups. Spot-level counts were then summed within each sample to generate pseudo-bulk counts per aggregate B cell population. Lowly expressed genes were filtered on a per-cluster basis using edgeR’s filterByExpr function. A quasi-likelihood negative binomial model was fitted with glmQLFit function, followed by hypothesis testing using glmQLFTest function under the contrast ‘Male versus Female’.

## Results

### Overview of the stPipe workflow

**Figure 1.**
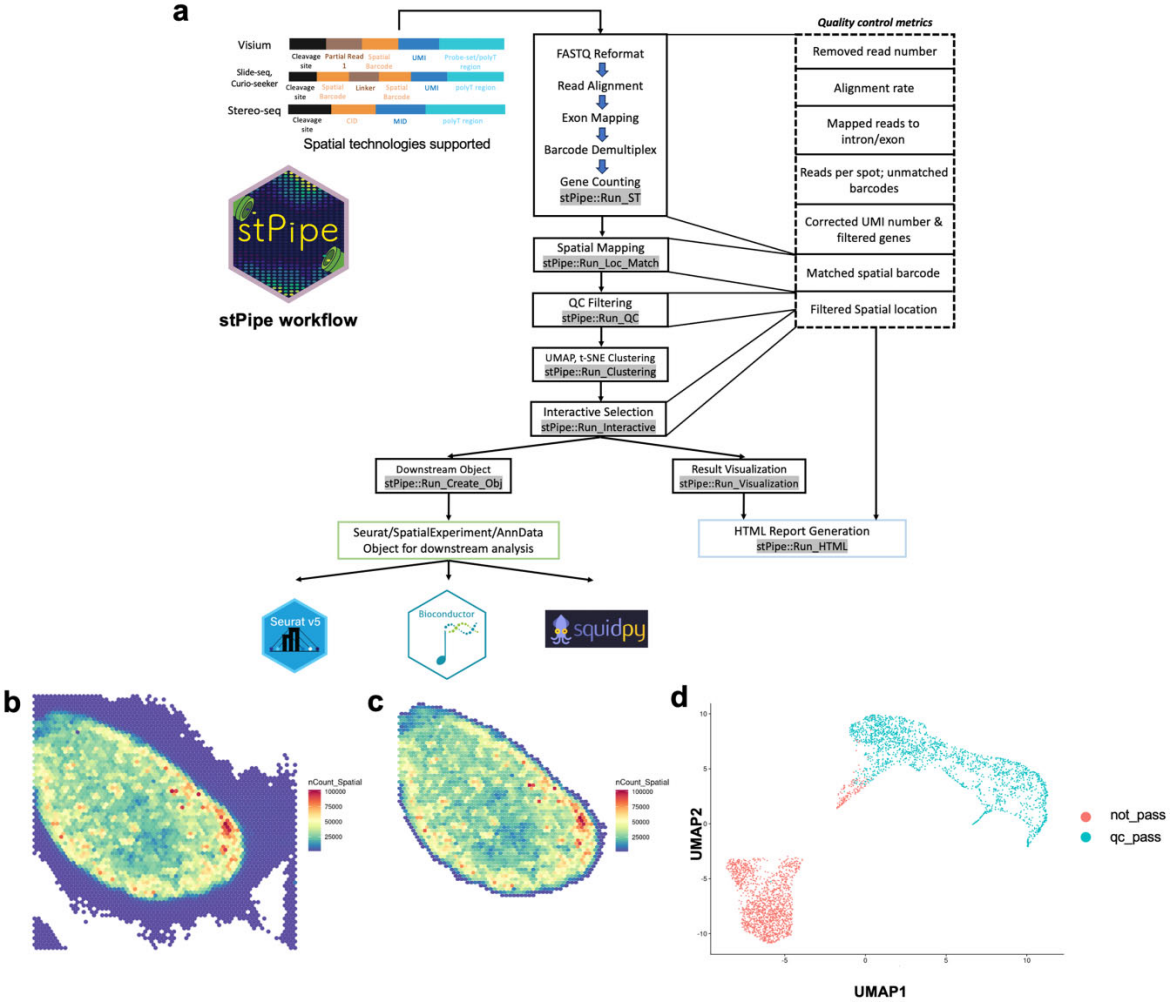
Overview of the stPipe workflow and sample QC. (**a**) The major steps in the stPipe data preprocessing pipeline are shown along with the QC statistics collected at each stage. The final output of the pipeline is a matrix of gene counts annotated with corresponding spatial information, sample annotations, and collected QC metrics during corresponding processing steps for use in downstream analysis and an HTML report that summarizes these data quality metrics. The resulting Seurat, SpatialExperiment, or Anndata object can be passed as input to Seurat or various Bioconductor packages which rely on SpatialExperiment objects, or a Squidpy workflow to perform further downstream analysis to explore the biological questions of interest. (**b**) Spatial heatmap of raw UMI count before the Run_QC function of FFPE, CA mouse spleen sample 709. (**c**) Spatial heatmap of raw UMI count after the Run_QC function is applied to this sample. The spatial heatmap suggests the tissue integrity of mouse spleen, as well as successful removal of background using the Run_QC function. (**d**) UMAP representation of the FFPE, CA, mouse spleen sample 709 after the Run_QC function is applied, spots pass QC filter and spots do not are projected as two colours. This suggests the removal of background from tissue.

stPipe is an R package that can handle data generated from popular sST protocols, including 10× Visium, BGI Stereo-seq, Slide-seq, and Curio-seeker (Fig. 1a). The pipeline initiates with PE FASTQ files and outputs a gene count matrix with matched spatial locations, QC statistics, clustering results, and a summary HTML report. stPipe re-uses and extends functionality from a number of other R- and Python-based packages, including scPipe for FASTQ reformatting, exon mapping, barcode demultiplexing, and gene counting; Rsubread for aligning reads to a reference genome or collection of probe-sets [20]; DropletUtils to perform QC filtering [21]; Shiny for the creation of a interactive region of interest (ROI) selection tool [22]; and Seurat [23], SpatialExperiment [24], and anndata [25] to organize the spatial counts and sample annotation information. Computationally intensive functions are implemented in C++ and wrapped as R functions using the Rcpp package [26]. A configuration file is required by stPipe to allow users to specify parameters related to the data type being analysed. For example, paths to FASTA and gff3 files are required for processing samples generated using poly-A-based protocols, and paths to h5 mapping files are needed for Stereo-seq-based data. The individual function help pages, as well as the stPipe package vignette, provide explanations of various configuration file parameters.

### Preprocessing sequencing data from ST with stPipe

The stPipe workflow (Fig. 1a) begins with the function Run_ST, which streamlines multiple steps into one cohesive process, including FASTQ or BAM file reformatting, read alignment, exon mapping, barcode demultiplexing, and gene counting. The Run_ST function builds upon the approach followed by scPipe, which has been further adapted and extended for the specific requirements of ST data analysis. Different sST protocols have different configurations of the read structure (Supplementary Fig. S1). Visium stores its 16-bp spatial barcode followed by 12-bp UMI in FASTQ read 1, while Slide-seq has an 8-bp spatial barcode, 18-bp bridge sequence, 8-bp spatial barcode (7-bp in Slide-seq V2) and 8-bp UMI (9-bp in Slide-seq V2) in FASTQ read 1. Initially, FASTQ or BAM (for Slide-seq-based experiments) files are reformatted, where barcodes and UMI information are extracted and incorporated into the read headers. For BGI Stereo-seq (STomics) data, this step involves the mapping and deconvolution of spatial barcodes to their corresponding x,y coordinate pairs as described in the provided mapping file for the chip. This is performed by a C++ script either run standalone or with the provided Run_ST function in the stPipe package using Rcpp [26]. Deconvolution is performed on the PE FASTQ files by translating the coordinate and UMI data in read 1 and inserting both into the header for read 2. The resulting FASTQ file is used in the subsequent stPipe preprocessing pipeline. Configuration for deconvolution is provided in the required config file including locations of the paired FASTQ files and the coordinate mapping file for the chip.

After reformatting, sequence alignment is performed using Rsubread, which maps the reads to a reference genome (or library of probe-sets for Visium probe-based platforms), resulting in a BAM file with positional information. Aligned reads are then assigned to annotated genomic features (e.g. exons or transcripts) using user-provided annotations in GTF or GFF format. Following alignment, exon mapping is performed to assign reads to specific exonic regions. The workflow also includes barcode demultiplexing to identify individual spatial location across the whole tissue section being captured. It is noteworthy that stPipe uses different strategies for probe-based and polyA-based protocols. The former uses a probe-set version to construct annotation files such as fa and GFF3 files, while the latter relies on the gene annotation files specified by the user.

Lastly, the Run_ST function generates a gene count matrix after performing UMI deduplication to remove polymerase chain reaction (PCR) duplicates. The deduplication approach in stPipe uses a distance-based method, comparing UMI sequences within a specified Hamming distance. When one UMI has significantly more reads than the other, the two are considered duplicates. This ensures accurate gene quantification by minimizing the impact of sequencing and PCR errors. Additionally, the Run_ST function implements parallel computing via the mclapply function in order to save computing time when processing batches of samples.

### Matching spatial and gene count information

After obtaining the gene count matrix with relevant sample index which refers to the spatial barcode sequence using the Run_ST function, the Run_Loc_Match function can be used to obtain spatial locations with corresponding UMI counts for different technologies. For 10× Visium, there are currently five different chip types, each of which has a fixed relationship between spatial barcodes and spatial coordinates. For Stereo-seq, Slide-seq, and Curio-seeker, barcodes are randomly distributed across spatial coordinates which must be mapped in a sample-specific way. For 10× Visium data, stPipe requires the user to specify the chip version and then uses internally stored 10× Visium coordinates to match the spatial barcodes. stPipe also provides additional pixel computation of each spot for Visium technology, which is achieved by circle detection based on Python OpenCV library [27]. For the remaining protocols, users are required to provide a path to the sample-specific spatial coordinate csv file.

### Quality assessment with stPipe

After matching the gene count profile with corresponding spatial locations, the Run_QC function can be used to filter out low-quality data (Fig. 1d). stPipe provides two different options for this: max_slope and EmptyDropletUtils. For the former approach, the filtering process is carried out based on the raw UMI count in each spatial location. Locations with counts that fall below a certain threshold are considered low quality and removed from the gene count matrix. This method retains only the spatial locations with significant transcriptomic signals to reduce noise from locations with minimal or no meaningful biological information. The threshold in this method is computed by analyzing the distribution of UMI counts across spatial locations, and identifying the point of maximum slope in the cumulative UMI distribution curve. This point often corresponds to the transition between background noise and biological signal. The second EmptyDropletUtils option makes use of the DropletUtils package, which offers a more sophisticated computation to identify spatial locations that contain real cells, as opposed to positions that contain ambient RNA or other noise. This method calculates a false discovery rate (FDR) to assess the likelihood of each spatial location containing one or more real cells. Filtering can be fine-tuned using both *P*-value and FDR thresholds, offering greater flexibility in distinguishing between noise and meaningful data. Spatial locations are retained if they meet the significance criteria for either the *P*-value or FDR. These two thresholds are specified in the stPipe config file.

### stPipe allows interactive selection of regions of interest

**Figure 2.**
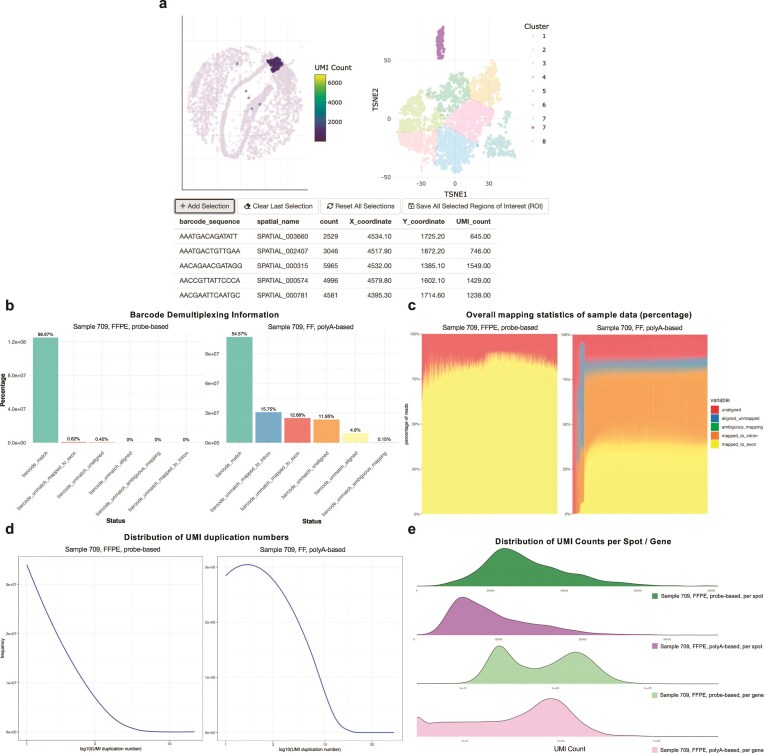
Interactive R-shiny web app created by the Run_Interactive function and visualization of QC metrics created by the Run_Visualization function for data quality assessment. (**a**) Visualization of Slide-seq mouse brain sample Puck_200115_08. The Run_Interactive function offers flexible options for selecting a ROI through four intuitive buttons. The ‘Add Selection’ button allows users to add spatial coordinates along with corresponding metadata, such as UMI count and spatial barcode sequences, each time an ROI is selected. The ‘Clear Last Selection’ button removes the most recently selected ROI from the current selection list. The ‘Reset All Selections’ button resets both the spatial heatmap and clustering plot, providing a clean slate for a new selection. Finally, the ‘Save All Selected ROI’ button saves the finalized selection as ‘selected_ROI’ object in the user’s R global environment, streamlining data management and export. In this example, the selection of cluster 7, highlighted in purple on the t-SNE plot, is found to mostly correspond to the choroid plexus region in the spatial UMI count plot. (**b**) Barplot showing spatial barcode demultiplexing information between 10× Visium probe-based (left) and polyA-based (right) protocols to assess sequencing accuracy. (**c**) Stacked bar plots showing the mapping rate, separated into reads that map to exons, introns, and those that are ambiguously mapped or map elsewhere in the genome (ordered by exon mapping rate) between 10× Visium probe-based (left) and polyA-based (right) protocols. (**d**) UMI duplication plot between a probe-based sample (left) with a higher UMI duplication number than a polyA-based one (right). A distribution skewed toward lower duplication values indicates higher library complexity and minimal redundancy, suggesting that the sequencing depth is well-matched to the diversity of the transcriptome. In contrast, a pronounced tail toward higher duplication values suggests substantial over-sequencing or PCR amplification biases, as many reads may originate from the same underlying transcript molecule. **(e)** UMI count distribution between sample 709 with two protocols, the first and last two are plotted as distribution of raw UMI count per spot and $\log _{10}$ UMI count per gene respectively.

The Run_QC function primarily acts as a tool for filtering out low-quality data; however, it may not effectively eliminate all background or out-of-tissue spots for the Visium platform with high precision. To address this challenge, the Run_Interactive function was implemented to allow users to more accurately select ROI, such as in-tissue spots for Visium data. This function is built based on the R shiny package [22] with embedded Plotly for enhanced interactivity, supporting various selection tools, including zoom, pan, rectangular selection, and lasso selection. It also enables linking of each spot’s spatial location to its corresponding point in the interactive UMAP (uniform manifold approximation and projection) or t-SNE (t-distributed stochastic neighbor embedding) plots provided by the Run_Clustering function. Prior to dimensionality reduction and clustering, highly variable genes (HVGs) are selected. After feature selection, the Run_Clustering function performs dimensionality reduction via UMAP and t-SNE, followed by clustering using methods such as Louvain, Leiden, or *k*-means. It thereby produces interactive plots wherein each spot’s spatial location is linked to its corresponding point in the UMAP or t-SNE embedding. This linkage facilitates an intuitive comparison between the reduced dimensional representation, the clustering outcomes, and the original spatial coordinates (Fig. 2a).

Users can either select the ROI directly on the spatial image, which is then mapped onto the UMAP or t-SNE plot, or select a specific cluster on the UMAP or t-SNE clustering map, with the selected cluster points mapped back to their corresponding spatial locations. In addition, the Run_Interactive function includes four interactive buttons, which include ‘Add Selection’, ‘Clear Last Selection’, ‘Reset All Selections’, and ‘Save All Selected ROI’, allowing users to easily manage their selections. As the selected ROIs are visually highlighted, users can precisely choose ROI and easily save the refined data back to the global environment in R, enhancing control and accuracy in data selection.

#### Data visualization and further quality control with stPipe

The Run_Visualization function visualizes both spatial-level and read-level information for the results generated in the previous steps of stPipe. This function outputs both raw and log-transformed UMI counts for spatial visualization in a heatmap format (Fig. 1 b and c), while demultiplexing results are shown in barplot format (Fig. 2b), mapping statistics as a stacked barplot (Fig. 2c), and UMI duplication number as a line plot (Fig. 2d). For multi-sample experiments, UMI counts distribution plots can be produced to assess data quality between samples (Fig. 2e). These metrics provide complementary insights into both spatial-level and read-level quality. For example, lower exon mapping rates or high ambiguity in alignment can indicate suboptimal RNA quality or technical issues during library preparation, while excessive PCR duplication revealed by UMI deduplication statistics may indicate low library complexity. The spatial UMI heatmap can flag tissue integrity issues or specific spatial patterns when compared to the corresponding H&E image. Overall, these QC metrics are valuable for flagging poor-quality samples or tissue regions to filter out before proceeding to downstream analysis.

#### Automatic HTML report generation

The Run_HTML function is designed to automatically generate an HTML report in R Markdown format. This function takes outputs from the Run_QC, Run_Visualization, and Run_Clustering functions and builds a report with plots summarizing barcode demultiplexing, UMI duplication, mapping statistics, QC thresholds, the number of spatial locations before and after the Run_QC step, and interactive t-SNE and UMAP plots with clustering results, each within its own subsection to illustrate its relevant importance within the pipeline. A key feature of stPipe is the collection of a consistent set of QC measures and data visualizations across different platforms, which facilitates comparisons between multiple datasets.

#### Organizing spatial counts for downstream analysis

Spatial count data is organized by the Run_Create_Obj function, which constructs flexible objects for downstream analysis across various mainstream workflows. Run_Create_Obj allows users to create: (i) R Seurat spatial objects to allow compatibility with Seurat sST workflows; (ii) R SpatialExperiment objects for use in Bioconductor sST workflows such as nnSVG for spatially variable gene (SVG) identification [28] and STdeconvolve [29] or SpatialDecon [30] for cell type inference and deconvolution; and (iii) Python AnnData spatial objects for use in Squidpy sST workflows [25].

#### stPipe’s config file helps manage the analysis workflow

stPipe simplifies the use of each function and overall code readability via a config file. The essential inputs in the config file include the data directory, output directory, sample species, technology version, specified spatial coordinate system, the format of the read structure, number of threads to process data over, number of reads to process, and the number of spatial locations. Other platform-specific information such as the spatial location csv file path for Slide-seq or Curio-seeker data, specific fa and gff files for polyA-based sequencing methods, tiff image path for pixel computation, path to h5 mapping file for Stereo-seq data, the QC method, and the ratio set for Run_QC function. When dealing with multiple samples, only those run using the same platform and species can be processed together in stPipe.

### Preprocessing with stPipe makes multi-sample analyses easier

Multiple samples (167, 168, 544, 545, 708, and 709) from the *SpatialBenchVisium* mouse spleen dataset [15] were preprocessed using stPipe. After preprocessing, cell type annotation was performed for each sample via spatial clustering using Seurat workflow (Supplementary Fig. S2a and b). Each sample contains over 10 million reads and takes under 30 min to process on a standard Linux server (with 4 CPUs and 24 GB RAM) using the current stPipe workflow for either the polyA-based or probe-based Visium platform. After spatial location matching and selection, the spots retained for each sample by stPipe shared over 98% intersection ratio with the 10× Space Ranger output. For each sample, UMI count and captured gene per spot had very similar distributions between stPipe and 10× Space Ranger outputs. For spleen sample 709, which was profiled across four different protocols (Supplementary Fig. S3a), differences between stPipe and 10× Space Ranger in terms of QC metrics including Percentage of Mapped Reads, Total Genes Detected, Median UMI Counts per Spot and Mean Reads Under Tissue per Spot were <1.5% on average, indicating that stPipe recovers comparable results to the vendor provided software.

The Run_Create_Obj function, followed by downstream analyses that included SVG detection, differential expression (DE) analysis, ROAST gene set testing [31], and cell type inference, followed the same workflow outlined in the original study by Du *et al.* [15]. The downstream results from gene-specific (SVG, DE) and cell-type-specific analyses further underscore the reliability of stPipe. Specifically, the *MA*-plot for the DE comparison of the B cell cluster (see Supplementary Fig. S2c) reveals a comparable trend in DE analysis to that reported in the original study, which was based on Space Ranger output, with the accurate detection of enrichment of sex-specific genes (those from the Y chromosome and genes on the X chromosome that escape X inactivation) that are expected to differ when comparing male versus female wild-type samples (ROAST *P*-value = .0005). These results affirm stPipe’s capability to consistently identify key biological features. In terms of SVG and HVG identification, *Car2* consistently emerged as the top-ranked gene in both categories, aligning with the findings of the original paper. Additionally, cell type inference results (Supplementary Fig. S3b) show comparable proportions, with an average difference of <2% across cell types when comparing stPipe with Space Ranger results. Both analyses employed an identical marker gene list, covering plasma cells, T cells, neutrophils, B cells, germinal centers, and erythrocytes, which underscores the robustness and consistency in cell type classification between these two preprocessing pipelines.

### stPipe preprocessing of Visium, Slide-seq, and Stereo-seq data enables comparisons across platforms

Publicly available datasets profiling mouse brain tissues across Visium, Slide-seq, and Stereo-seq technologies were preprocessed from raw FASTQ or BAM files into gene count matrices using stPipe with corresponding workflows implemented in the Run_ST function. Preprocessing these data on a standard Linux server (with 4 CPUs and 24 GB RAM) took below 30 min for 65 million PE reads from the Visium sample, 2 h for over 200 million PE reads from the Slide-seq sample, and 8 h for the 270 million PE reads from the Stereo-seq sample. Next, the Run_Loc_Match function was used to obtain matched spatial location information. Finally, Seurat spatial objects were constructed via the Run_Create_Obj function, followed by downstream analyses which includes marker gene identification, and inference of both cell types and spatial domains using the Seurat workflow.

In the mouse brain, the hippocampal region CA2 is distinguished from the CA1 and CA3 regions by several unique characteristics, such as its distinct gene expression profile, lack of long-term potentiation, and higher resistance to cell death. However, due to the hippocampus’s curved structure, accurately locating CA2 relative to CA1 and CA3 along the transverse axis within coronal and sagittal slices, as shown in the Allen Brain Atlas [32], can be challenging. Hence, the result of spatial domain identification of the CA2 region with related significant marker genes can be considered as an important indicator of stPipe’s preprocessing performance. In addition to inferring cell types and spatial domains, marker gene sensitivity tests have been performed to validate these inference outcomes via the FindMarkers function implemented in the Seurat package with a significance criteria of log-fold change >0.25 and adjusted *P*-value < .01. Domain-specific marker genes include *Hpca, Prox1, Ptgds, Ttr, Prdm8*, and *Slc17a7* for the hippocampus region and region-specific marker genes including *Rgs14, Camk4, Amigo2*, and *Ntf3* for the CA2 region were used for evaluation.

**Figure 3.**
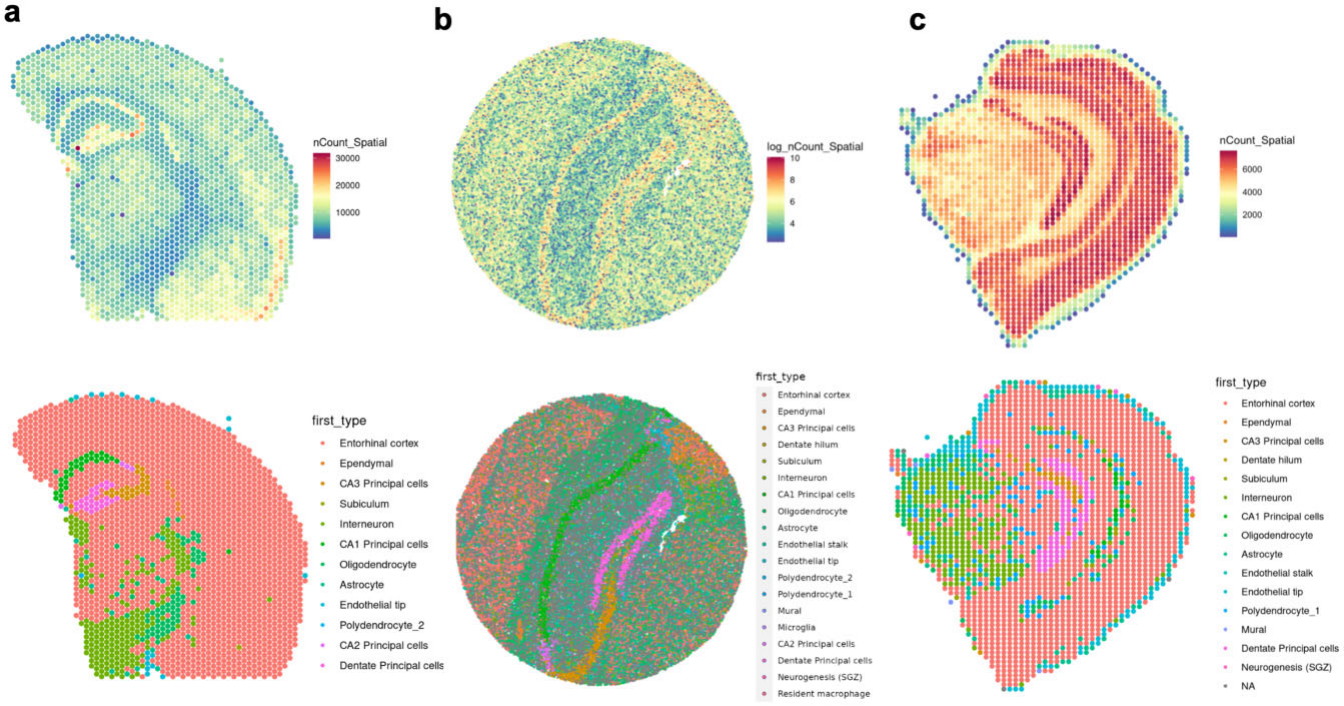
Spatial UMI plot and deconvolution result of mouse brain across different platforms. (**a**) Visualization of mouse brain sample based on the 10× Visium platform. The upper panel depicts the spatial UMI distribution following preprocessing with the stPipe workflow, and the lower panel illustrates the deconvolution results obtained using the RCTD algorithm implemented in the Seurat workflow. (**b**) Visualization of mouse brain sample processed based on Slide-seq platform, following the same figure structure and workflow as in panel (a). (**c**) Visualization of mouse brain sample processed based on Stereo-seq platform, following the same figure structure and workflow as in panel (a). Further details regarding the datasets and analyses are provided in the dataset description and analysis sections, respectively.

Among these platforms, Visium (Fig. 3a) offers lower spatial resolution but captures broad regional differences, making it suitable for identifying general spatial patterns; Slide-seq (Fig. 3b) improves upon resolution, enabling the identification of finer hippocampus structures with the CA2 region inside it; Stereo-seq (Fig. 3c) provides the highest resolution among the three platforms, effectively distinguishing sub-regional domains, such as the CA1 and CA3 regions, but it fails to identify the CA2 region accurately. For marker gene sensitivity testing, most of the significant domain-specific and region-specific marker genes were consistently captured by three platforms for most regions, except for Visium and Stereo-seq which failed to detect CA2 marker genes across the captured spatial section.

## Discussion

Despite rapid growth of the ST field across a diverse range of technology platforms and data analysis tools tailored to spatial analysis, limited options exist for handling the raw data obtained from mainstream sST protocols. The stPipe package bridges this gap, accepting raw FASTQ files and allowing flexibility in read structure to obtain spatial gene counts from various sST protocols, including 10× Visium, Slide-seq, and Stereo-seq. stPipe outputs numerous QC metrics obtained during the data preprocessing workflow and presents these results in an HTML report to assist users in quality assessment and downstream analysis. In terms of system requirements, stPipe can be run with modest resources and is computationally efficient, as evidenced by its ability to complete the preprocessing of a typical Visium sample with 80 million PE FASTQ reads using 8 CPUs and 24GB RAM in around 20 minutes. Compared to other vendor-provided preprocessing tools, stPipe supports data across multiple technology platforms and is highly customizable (Supplementary Table S2).

The application of stPipe to a multi-sample mouse spleen dataset (*SpatialBenchVisium*) revealed high concordance with the 10× Space Ranger results in terms of the spatial features detected and very similar distributions of UMI counts and genes captured per spatial location between the two software. In addition, the identification of SVG and HVG, annotated cell type proportions, and ability to detect the enrichment of sex-specific gene signatures were all in line with previously published results and the expected biological ground truth. We also provided a practical use case of stPipe in benchmarking a mouse brain dataset across three mainstream platforms. This analysis demonstrated how stPipe can be seamlessly integrated with downstream analytic tools to produce accurate cell-type annotations corresponding to known anatomical clusters and used to benchmark the ability of different platforms to recover the CA1, CA2, and CA3 hippocampal subfields.

Planned future improvements to and applications of stPipe are in the following three areas: (i) extending support to various downstream analysis tasks such as SVG identification, deconvolution/cell type inference, marker gene detection, and cell–cell communication based on the results obtained from preprocessing; (ii) extending support to recently released sST platforms such as 10× Visium HD, accommodating new formats for spatial count data like SpatialData [33], and other tools to improve preprocessing results such as SpotClean [34], which corrects for spot swapping to improve the accuracy of gene-specific UMI counts; and (iii) applying stPipe in larger-scale sST benchmarking analyses.

## Supplementary Material

lqaf167_Supplemental_File

## Data Availability

The stPipe package is available from Bioconductor (https://bioconductor.org/packages/stPipe) and Zenodo (https://doi.org/10.5281/zenodo.17430088). Analysis code for the stPipe workflow described in the ‘Results’ section above is available in the package vignette, and the remaining analysis code for this paper is available from Zenodo at https://doi.org/10.5281/zenodo.17430088. A vignette accompanying the package provides further details on implementation and an example use case on the downsampled 10× Visium FFPE, CytAssist processed mouse spleen sample 709 from the SpatialBenchVisium dataset [15].

## References

[1] Marx V . Method of the year: spatially resolved transcriptomics. Nat Methods. 2021;18:9–14. 10.1038/s41592-020-01033-y. 33408395

[2] Wang Y, Liu B, Zhao G et al. Spatial transcriptomics: technologies, applications and experimental considerations. Genomics. 2023;115:110671. 10.1016/j.ygeno.2023.110671. 37353093 PMC10571167

[3] Moses L, Pachter L. Museum of spatial transcriptomics. Nat Methods. 2022;19:534–46. 10.1038/s41592-022-01409-2. 35273392

[4] Tian L, Chen F, Macosko EZ. The expanding vistas of spatial transcriptomics. Nat Biotechnol. 2023;41:773–82. 10.1038/s41587-022-01448-2. 36192637 PMC10091579

[5] Ståhl PL, Salmén F, Vickovic S et al. Visualization and analysis of gene expression in tissue sections by spatial transcriptomics. Science. 2016;353:78–82. 10.1126/science.aaf2403. 27365449

[6] Oliveira MF, Romero JP, Chung M et al. High-definition spatial transcriptomic profiling of immune cell populations in colorectal cancer. Nat Genet. 2025;57:1512–23. 10.1038/s41588-025-02193-3. 40473992 PMC12165841

[7] Chen A, Liao S, Cheng M et al. Spatiotemporal transcriptomic atlas of mouse organogenesis using DNA nanoball-patterned arrays. Cell. 2022;185:1777–92. 35512705 10.1016/j.cell.2022.04.003

[8] Rodriques SG, Stickels RR, Goeva A et al. Slide-seq: a scalable technology for measuring genome-wide expression at high spatial resolution. Science. 2019;363:1463–7. 30923225 10.1126/science.aaw1219 PMC6927209

[9] Stickels RR, Murray E, Kumar P et al. Highly sensitive spatial transcriptomics at near-cellular resolution with Slide-seqV2. Nat Biotechnol. 2021;39:313–9. 33288904 10.1038/s41587-020-0739-1 PMC8606189

[10] Janesick A, Shelansky R, Gottscho AD et al. High resolution mapping of the tumor microenvironment using integrated single-cell, spatial and *in situ* analysis. Nat Commun. 2023;14:8353. 38114474 10.1038/s41467-023-43458-x PMC10730913

[11] Gong C, Li S, Wang L et al. SAW: an efficient and accurate data analysis workflow for Stereo-seq spatial transcriptomics. GigaByte. 2024;2024:gigabyte111. 10.46471/gigabyte.111 10.46471/gigabyte.111. 38434930 PMC10905255

[12] Curio Bioscience . Seeker Spatial Transcriptomics Kits. https://curiobioscience.com/seeker/ (30 September 2025, date last accessed).

[13] Sztanka-Toth TR, Jens M, Karaiskos N et al. Spacemake: processing and analysis of large-scale spatial transcriptomics data. Gigascience. 2022;11:giac064. 10.1093/gigascience/giac064. 35852420 PMC9295369

[14] Tian L, Su S, Dong X et al. scPipe: a flexible R/Bioconductor preprocessing pipeline for single-cell RNA-sequencing data. PLoS Comput Biol. 2018;14:e1006361. 10.1371/journal.pcbi.1006361. 30096152 PMC6105007

[15] Du MR, Wang C, Law CW et al. Benchmarking spatial transcriptomics technologies with the multi-sample SpatialBenchVisium dataset. Genome Biol. 2025;26:77. 10.1186/s13059-025-03543-4. 40156041 PMC11954323

[16] Cable DM, Murray E, Zou LS et al. Robust decomposition of cell type mixtures in spatial transcriptomics. Nat Biotechnol. 2022;40:517–26. 10.1038/s41587-021-00830-w. 33603203 PMC8606190

[17] Saunders A, Macosko EZ, Wysoker A et al. Molecular diversity and specializations among the cells of the adult mouse brain. Cell. 2018;174:1015–30. 10.1016/j.cell.2018.07.028. 30096299 PMC6447408

[18] Ritchie ME, Phipson B, Wu D et al. limma powers differential expression analyses for RNA-sequencing and microarray studies. Nucleic Acids Res. 2015;43:e47. 10.1093/nar/gkv007. 25605792 PMC4402510

[19] Robinson MD, McCarthy DJ, Smyth GK. edgeR: a Bioconductor package for differential expression analysis of digital gene expression data. Bioinformatics. 2010;26:139–40. 10.1093/bioinformatics/btp616. 19910308 PMC2796818

[20] Liao Y, Smyth GK, Shi W. The Subread aligner: fast, accurate and scalable read mapping by seed-and-vote. Nucleic Acids Res. 2013;41:e108. 10.1093/nar/gkt214. 23558742 PMC3664803

[21] Lun AT, Riesenfeld S, Andrews T et al. EmptyDrops: distinguishing cells from empty droplets in droplet-based single-cell RNA sequencing data. Genome Biol. 2019;20:1–9. 10.1186/s13059-019-1662-y. 30902100 PMC6431044

[22] Chang W, Cheng J, Allaire J et al. Shiny: web application framework for R. R package version. 2017;1:2017.

[23] Hao Y, Stuart T, Kowalski MH et al. Dictionary learning for integrative, multimodal and scalable single-cell analysis. Nat Biotechnol. 2024;42:293–304. 10.1038/s41587-023-01767-y. 37231261 PMC10928517

[24] Righelli D, Weber LM, Crowell HL et al. SpatialExperiment: infrastructure for spatially-resolved transcriptomics data in R using Bioconductor. Bioinformatics. 2022;38:3128–31. 10.1093/bioinformatics/btac299. 35482478 PMC9154247

[25] Palla G, Spitzer H, Klein M et al. Squidpy: a scalable framework for spatial omics analysis. Nat Methods. 2022;19:171–8. 10.1038/s41592-021-01358-2. 35102346 PMC8828470

[26] Eddelbuettel D, François R. Rcpp: Seamless R and C++ integration. J Stat Softw. 2011;40:1–18. 10.18637/jss.v040.i08.

[27] Culjak I, Abram D, Pribanic T et al. A brief introduction to OpenCV. In: 2012 proceedings of the 35th international convention MIPRO. IEEE, 2012, 1725–30.

[28] Weber LM, Saha A, Datta A et al. nnSVG for the scalable identification of spatially variable genes using nearest-neighbor Gaussian processes. Nat Commun. 2023;14:4059. 10.1038/s41467-023-39748-z. 37429865 PMC10333391

[29] Miller BF, Huang F, Atta L et al. Reference-free cell type deconvolution of multi-cellular pixel-resolution spatially resolved transcriptomics data. Nat Commun. 2022;13:2339. 10.1038/s41467-022-30033-z. 35487922 PMC9055051

[30] Danaher P, Kim Y, Nelson B et al. Advances in mixed cell deconvolution enable quantification of cell types in spatial transcriptomic data. Nat Commun. 2022;13:385. 10.1038/s41467-022-28020-5. 35046414 PMC8770643

[31] Wu D, Lim E, Vaillant F et al. ROAST: rotation gene set tests for complex microarray experiments. Bioinformatics. 2010;26:2176–82. 10.1093/bioinformatics/btq401. 20610611 PMC2922896

[32] Wang Q, Ding SL, Li Y et al. The Allen mouse brain common coordinate framework: a 3D reference atlas. Cell. 2020;181:936–53. 10.1016/j.cell.2020.04.007. 32386544 PMC8152789

[33] Marconato L, Palla G, Yamauchi KA et al. SpatialData: an open and universal data framework for spatial omics. Nat Methods. 2025;22:58–62. 10.1038/s41592-024-02212-x. 38509327 PMC11725494

[34] Ni Z, Prasad A, Chen S et al. SpotClean adjusts for spot swapping in spatial transcriptomics data. Nat Commun. 2022;13:2971. 10.1038/s41467-022-30587-y. 35624112 PMC9142522

