# Supplementary material for: stPipe: a flexible and streamlined R/Bioconductor pipeline for preprocessing sequencing-based spatial transcriptomics data
Source: NAR Genom Bioinform. 2025 Nov 22;7(4):lqaf167. doi: 10.1093/nargab/lqaf167 (PMC12639247; doi:10.1093/nargab/lqaf167)
Supplement: lqaf167_Supplemental_File [file lqaf167_supplemental_file.pdf]

Supplementary Tables and Figures: *stPipe:* A flexible and streamlined R/Bioconductor pipeline for preprocessing sequencing-based spatial transcriptomics data

Supplementary Tables

1 List of marker genes used to annotate spatial clusters as different cell types in the 10x Visium mouse spleen data. 2
2 Summary table comparing sST preprocessing tools. 3

Supplementary Figures

1 Sequence configuration details for different sST platforms. 4
2 Cell type annotation for mouse spleen. 5
3 Comparison between stPipe and Space Ranger QC metrics and downstream cell type annotation results. 6

**Cell type/splenic region**	**Selected marker genes**
B cell	*Cd19, Cd22, Ighd, Cd5*
T cell	*Trac, Cd3d, Cd4, Cd3e, Cd8a*
Macrophage	*Cd274, Marco, Csf1r, Adgre1, Cd209b, Cd206, Cd80, Mac1, Cd68*
Neutrophil	*S100a9, S100a8, Ngp*
Erythrocyte	*Car2, Car1, Klf1*
Plasma cell	*Cd38, Cd138, Xbp1, Irf4, Prdm1, Cd27, Cd319, Mum1*
Germinal centre	*Cxcr4, Cd83, Bcl6, Rgs13, Aicda*
Marginal zone	*Marco, Lyz2, Ighd, Igfbp7, Igfbp3, Ly6d*

Supplementary Table 1. List of marker genes for different cell types or broader tissue regions expected in the mouse spleen selected from previous studies and existing literature. These marker genes were used to compute a "cluster score," calculated as the $\log_2$fold-change in expression between each spatial cluster and all other clusters. This quantitative measure was used to inform the cell type annotation of the spatial clusters.

Software Feature	Space Ranger	SAW	Curio Seeker	stPipe
1. Open-source	✗	✗	✓	✓
2. Platform-agnostic	✗	✗	✗	✓
3. Customisable processing mode	✗	✗	✗	✓
4. H&E image integration	✓	✗	✗	✓
5. Interactivity	✓	✗	✗	✓

Supplementary Table 2. Summary table comparing the features of different sST preprocessing tools. Features compared include: (1) whether the software is open-source; (2) is the software platform-specific, or able to handle raw data from multiple sST technologies; (3) can the software be customised to meet different user needs such as custom read configurations; (4) does the software support H&E image computation; and (5) does the software provide interactive modules to enhance usability.

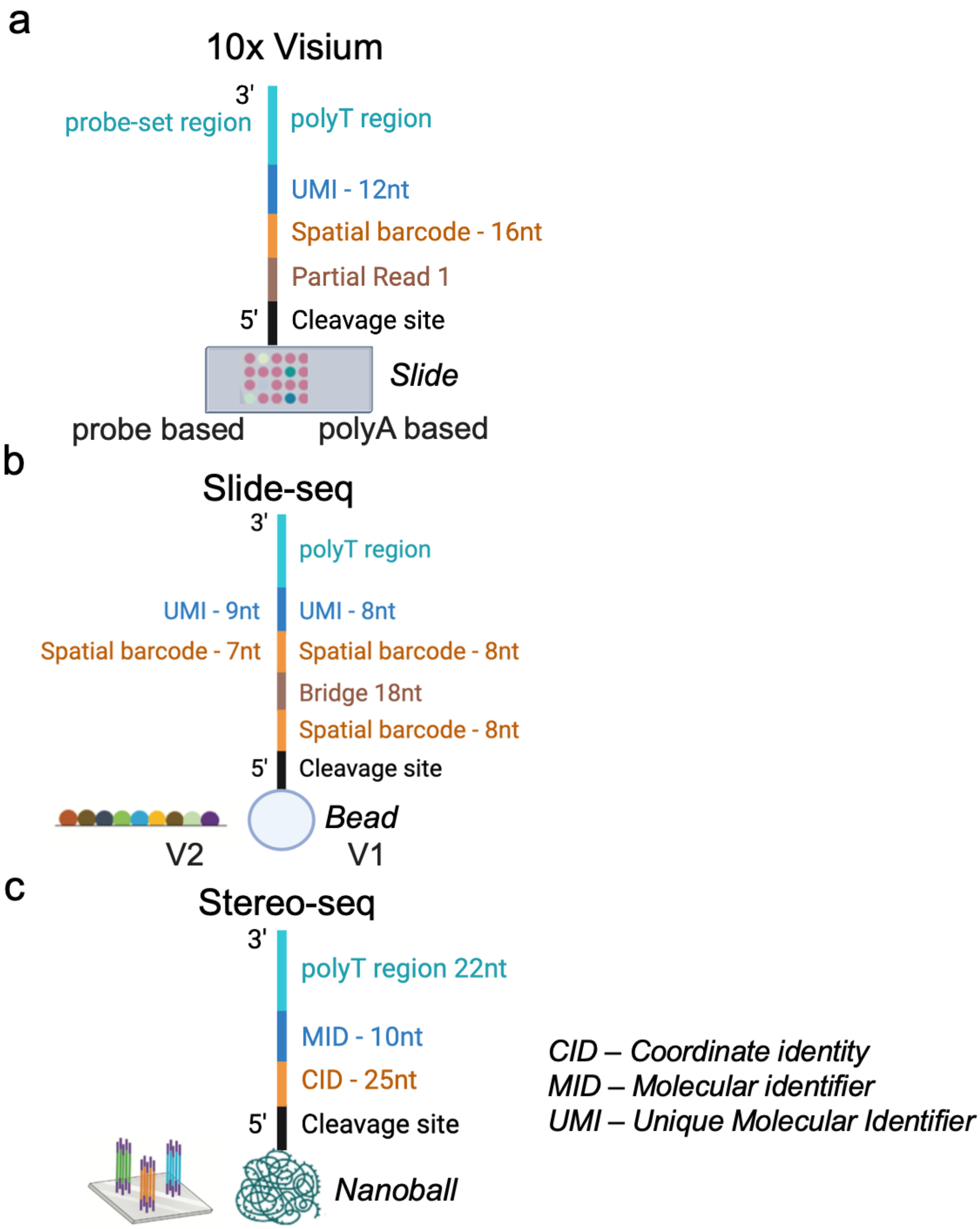

Supplementary Figure 1. Sequence configuration details for FASTQ files from different sST platforms, including 10x Visium, Slide-seq, and BGI Stereo-seq.

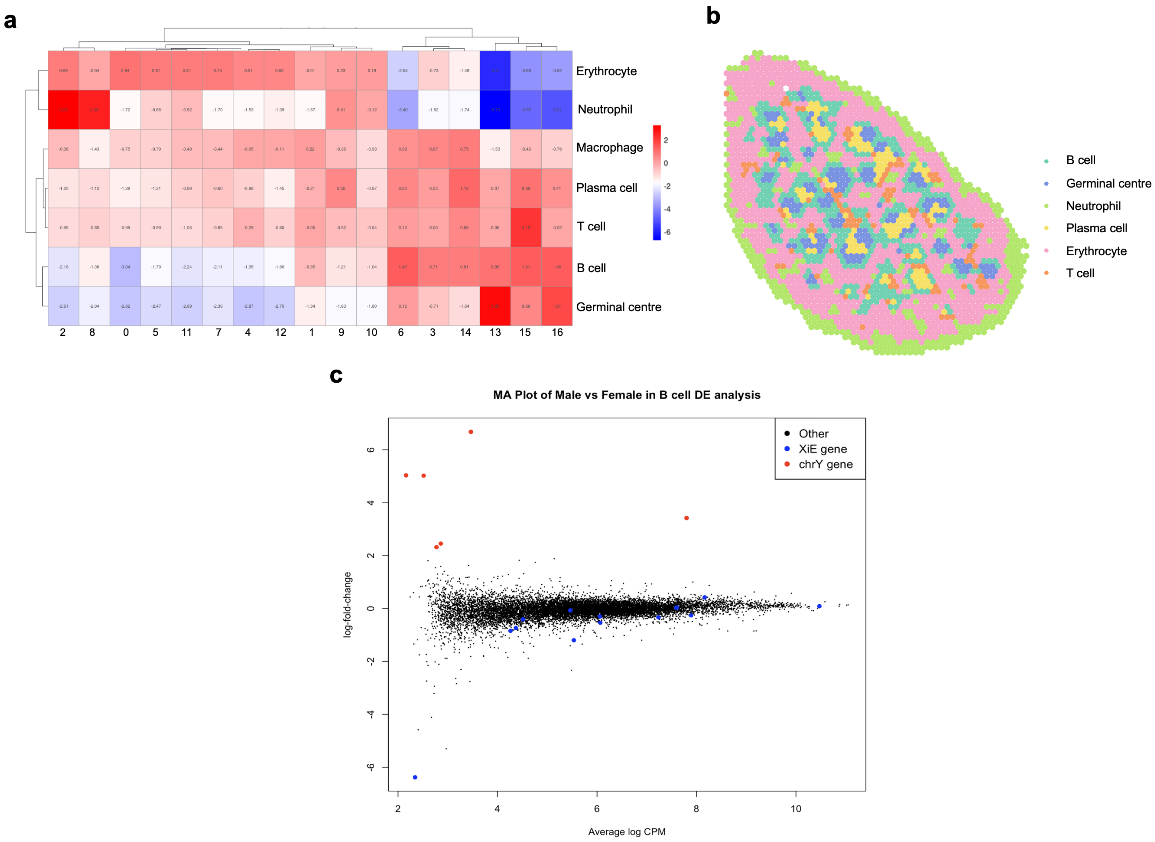

Supplementary Figure 2. Plot showing the intermediate results of analysing the 10x Visium mouse spleen data. (a) Heatmap of spatial cluster number vs. cell type with corresponding log-2 fold-change obtained from marker genes (Supplementary Table 1); (b) Spatial plot of mouse spleen, showing the distribution of B cells, Germinal centres, Neutrophils, Plasma cells, Erythrocytes, and T cells. (c) MA-plot showing sex-specific genes expected to be differentially expressed between male and female samples in the B cell cluster. These include genes on the X chromosome that escape X inactivation in female samples (blue points) and chromosome Y genes which are only present in male samples (red points).

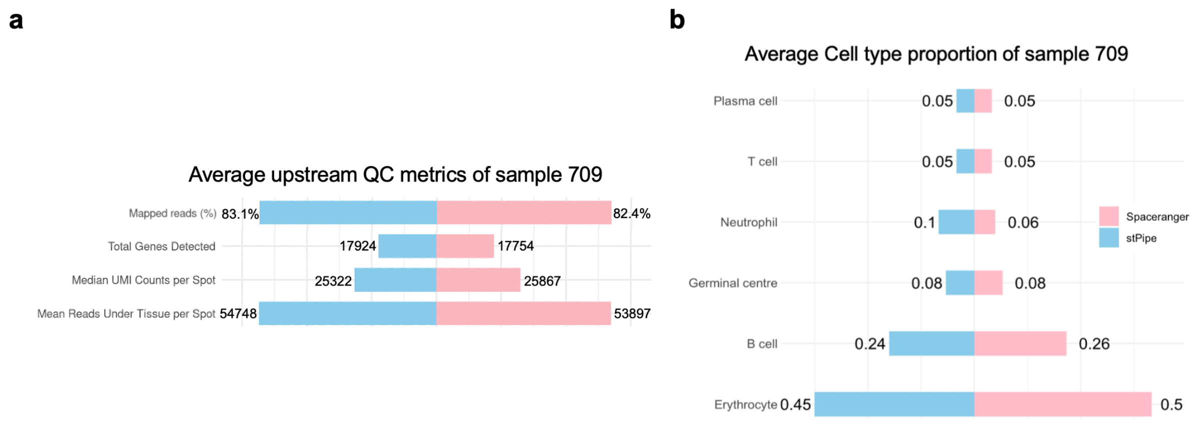

Supplementary Figure 3. Comparison between `stPipe` and `Space Ranger` QC metrics and downstream cell type annotation results. (a) Plot showing the averaged QC metrics including Percentage of Mapped Reads, Total Genes Detected, Median UMI Counts per Spot and Mean Reads Under Tissue per Spot from mouse spleen sample 709 across four Visium protocols for `Space Ranger` (pink) and `stPipe` (light blue). (b) Plot showing the averaged cell type proportions for mouse spleen sample 709 across four Visium protocols, grouped by cell type on the y-axis for `Space Ranger` (pink) and `stPipe` (light blue).
